# ASB17 Facilitates the Burst of LPS-Induced Inflammation Through Maintaining TRAF6 Stability

**DOI:** 10.3389/fcimb.2022.759077

**Published:** 2022-01-31

**Authors:** Pin Wan, Ge Yang, Simeng Zhang, Yaru Zhang, Yaling Jia, Xu Che, Zhen Luo, Pan Pan, Geng Li, Xulin Chen, Qiwei Zhang, Wen Zhang, Qiuping Tan, Yongkui Li, Jianguo Wu

**Affiliations:** ^1^ Guangdong Provincial Key Laboratory of Virology, Institute of Medical Microbiology, Jinan University, Guangzhou, China; ^2^ State Key Laboratory of Virology, College of Life Sciences, Wuhan University, Wuhan, China; ^3^ Foshan Institute of Medical Microbiology, Foshan, China; ^4^ The First Affiliated Hospital of Jinan University, Jinan University, Guangzhou, China; ^5^ Guangdong Longfan Biological Science and Technology, Foshan, China

**Keywords:** ASB17, NF-κB, TRAF6, K48-linked polyubiquitination, inflammatory cytokine

## Abstract

ASB17, a member of the ankyrin repeat and SOCS box-containing protein (ASB) family, has been supposed to act as an E3 ubiquitin ligase. Actually, little is known about its biological function. In this study, we found that ASB17 knocking-out impaired the expression of the pro-inflammatory cytokines CCL2 and IL-6 in bone marrow-derived dendritic cells (BMDCs) stimulated by lipopolysaccharide (LPS), indicating an inflammation-promoting role of this gene. We reveal that ASB17 promotes LPS-induced nuclear factor kappa B (NF-κB) signal activation through interacting with TNF receptor-associated factor 6 (TRAF6) which is a crucial adaptor protein downstream of toll-like receptors (TLR). ASB17 *via* its aa177–250 segment interacts with the Zn finger domain of TRAF6. The interaction of ASB17 stabilizes TRAF6 protein through inhibiting K48-linked TRAF6 polyubiquitination. Therefore, we suggest that ASB17 facilitates LPS-induced NF-κB activation by maintaining TRAF6 protein stability. The inflammation enhancer role of ASB17 is recognized here, which provides new understanding of the activation process of inflammation and immune response.

## Introduction

The ankyrin repeat and suppressor of cytokine signaling (SOCS) box-containing protein (ASB) family containing 18 members has been identified as an E3 ubiquitin ligase family (Kohroki et al., 2005; Liu et al., 2019). ASB1, ASB2, and ASB12 have been found to form complexes respectively with Cullin5–Rbx2 and have E3 Ub ligase activity (Kohroki et al., 2005). ASB7 ubiquitinates DDA3 for degradation to regulate spindle dynamics and genome integrity (Uematsu et al., 2016). ASB9 targets ubiquitous mitochondrial creatine kinase (uMtCK) and negatively regulates cell growth (Kwon et al., 2010). ASB11 mediates BIK ubiquitination and degradation to determine cell fate during different cellular stresses (Chen et al., 2019). Like other members of the ASB family, ASB17 protein contains two ankyrin repeats and one SOCS box (Guo et al., 2004). We recently reported that ASB17 mediates cell apoptosis in the testis by ubiquitylating and degrading BCLW and MCL1 (Yang et al., 2021). Through a study with ASB17 KO mice, we found that ASB17 was involved in cytokine regulation, so its biological function in inflammatory signaling was investigated in this work. Importantly, here we report a novel distinguishing activity of ASB17 showing that it functions as a ubiquitination inhibitor in regulation of the NF-κB signal pathway.

Cellular inflammatory signals are generally activated by microbial components *via* pattern recognition receptors (PRRs) such as toll-like receptors (TLRs), retinoid acid-inducible gene-I (RIG-I)-like receptors, and DNA-recognizing receptors (Kawai and Akira, 2010). TNF receptors and the interleukin-1 receptor (IL-1R) activate the subsequent downstream signaling of inflammation when cytokine ligands are induced (Gabay et al., 2010; Hayden and Ghosh, 2014). Tumor necrosis factor (TNF) receptor-associated factor 6 (TRAF6) functions as a crucial adaptor protein that mediates signaling events from the TLR family, IL-1 receptor, and TNF receptor superfamily (Cao et al., 1996; Chung et al., 2007). Thus, TRAF6 play important roles in the activation process of inflammation and immune response. When these receptors are triggered by their ligands, they activate TRAF6 to exert its E3 Ub ligase activity. TRAF6 forms a ubiquitin-binding enzyme complex with Ubc13 and Uev1A to attach lysine 63 (K63)–linked polyubiquitin chains to the substrates (Deng et al., 2000). TRAF6 also attaches K63-linked polyubiquitin chains to itself, which is required for triggering the activation of canonical NF-κB (Lamothe et al., 2008). TRAF6 attached with K63-linked polyubiquitin can recruit TAK1 and phosphorylate the IkB kinase complex IKKα/β/γ, leading to NF-κB activation (Deng et al., 2000). The TRAF6-mediated NF-κB signal pathway is involved in multiple pathological processes and especially essential for inflammatory diseases, which makes TRAF6 become a key modulating target of inflammation (Dainichi et al., 2019). TRAF6 can be ubiquitylated by a K48-linked polyubiquitin form, which leads to its proteasomal degradation. BICP0 and TRIM38 negatively regulate TRAF6-mediated NF-κB by promoting the K48-linked ubiquitination and degradation of TRAF6 (Zhao et al., 2012; Cao et al., 2019). Given the significant relevance of TRAF6-mediated NF-κB in inflammation activation, the modulation on TRAF6 can determine the pathological process of many inflammatory diseases (Du et al., 2017; Lv et al., 2018; Matsumoto et al., 2018; Wu et al., 2020). In this study, we recognize ASB17 as a TRAF6-stabilizing factor which enhances LPS-induced NF-κB activation. ASB17 facilitates the induction of pro-inflammatory cytokines *via* suppressing TRAF6 K48-linked ubiquitination. These will provide more knowledge about the TRAF6-mediated NF-κB signal pathway.

## Materials and Methods

### Animal Study

*Asb17* deficiency mice with the *Asb17* Exon 1 (1,595 bp) deleted were reserved by our laboratory (Yang et al., 2021). All animal experiments were approved by the Institutional Animal Care and Use Committee (IACUC) of the College of Life Sciences, Wuhan University (permit number: WDSKY0201901).

### Cells

THP-1 (human myeloid leukemia mononuclear cell line) and HEK293T (human embryonic kidney cell line) were purchased from the China Center of Type Culture Collection (CCTCC) (Wuhan, China). HEK293T cells were cultured in DMEM purchased from Gibco (Grand Island, NY, USA) supplemented with 10% fetal bovine serum (FBS), 100 U/ml penicillin, and 100 μg/ml streptomycin sulfate. THP-1 cells were cultured in RPMI 1640 and purchased from Gibco supplemented with 10% FBS, 100 U/ml penicillin, and 100 μg/ml streptomycin sulfate. Bone marrow-derived dendritic cells (BMDCs) were isolated from the femoral and tibia bone marrow of 6–8-week-old mice. Briefly, the bone marrow was flushed with RPMI 1640. Extracted cells were resuspended and passed through a 200-pore-sized mesh. Collected cells were resuspended in BMDC culture medium, which was made from RPMI 1640 medium containing 10% FBS, 100 U/ml streptomycin, 100 U/ml penicillin, and 20 ng/ml recombinant mouse granulocyte macrophage-colony stimulating factor (GM-CSF) in a 100-mm Petri dish. On the third day, 10 ml BMDC culture medium was added into the Petri dish. On the sixth and eighth days, the medium was changed in half: the old culture medium was collected and centrifuged, and the cell pellet was resuspended in the complete medium containing 20 ng/ml recombinant mouse GM-CSF, and then the cell suspension was returned to the original dish. On the tenth day, the culture medium was gently pipetted to collect the suspended cells and centrifuged at room temperature. The supernatant was discarded, while the cell pellet was resuspended in complete medium containing 10 ng/ml recombinant mouse GM-CSF and then spread on a cell culture plate at 37°C and 5% CO_2._

Bone marrow-derived macrophages (BMDMs) were isolated from the bone marrow of 6–8-week-old mice; these experiments were performed as described previously (Wan et al., 2019). Briefly, the bone marrow was flushed with RPMI 1640. Collected cells were resuspended and passed through a 200-pore sized mesh. Collected cells were resuspended with Red Blood Cell Lysis Buffer for 5 min, then collected cells were cultured in DMEM complemented with 10% FBS, 10%–20% L929 cell-conditioned medium, 100 μg/ml streptomycin sulfate, and 100 U/ml penicillin for 5–6 days.

### Reagents

Lipopolysaccharide (LPS) (Cat# L2630) and polybrene (Cat# TR-1003-G) were purchased from Sigma-Aldrich (St. Louis, MO, USA). Puromycin (Cat# ant-pr-1) was purchased from InvivoGene Biotech Co., Ltd. (San Diego, CA, USA). Cycloheximide (CHX) was purchased from Selleck (Houston, TX, USA). Protease Inhibitor Cocktail Tablets were purchased from Roche (Indianapolis, IN, USA). TRIzol reagent and Lipofectamine 2000 transfection reagent (Cat# 11668019) were purchased from Invitrogen (Carlsbad, CA, USA). Mouse GM-CSF (Cat#315-03) were purchased from PeproTech (Rocky Hill, NJ, USA).

### Antibodies

Anti-IRAK1 (D51G7) (Cat#4504), anti-TRAF6 (D21G3) (Cat#8028), anti-Myc (9B11) (Cat#2276), anti-ubiquitin (P4D1) (Cat#3936), anti-K48-linkage-specific polyubiquitin (D9D5) (Cat#8081), anti-K63-linkage-specific polyubiquitin (D7A11) (Cat#5621), anti-p65 (Cat#8242), and anti-phospho-p65 (Ser536) (Cat#3033) were purchased from Cell Signaling Technology (Beverly, MA, USA).Anti-TRAF6 (Cat#ab137452) was purchased from Abcam (Cambridge, MA, USA). Anti-Flag (Cat# F3165) and anti-HA (Cat# H6908) antibodies were purchased from Sigma-Aldrich. Anti-TRAF6 (Cat# 66498-1-lg) and anti-GAPDH (Cat# 60004-1-lg) were purchased from Proteintech (Wuhan, Hubei, China). Anti-Rabbit IgG FITC (Cat# A22120) and anti-Mouse IgG DyLight 649 (Cat# A23610) antibodies were purchased from Abbkine.

### Plasmids and Constructions

Candidate genes were cloned into pcDNA3.1(+)-3Flag vector or pCAGGS-HA vector. TRAF2, TRAF3, TRAF5, TRAF6, IRF7, STING, IRF3, TBK1, and ASB17 were cloned into the pcDNA3.1(+)-3Flag vector. NLRP3-PYD, ASB17, and NLRP3 were cloned into the pCAGGS-HA vector. NLRP3 point mutant and truncated ASB17 genes were cloned into the pcDNA3.1-3Flag vector. All recombinant plasmids were confirmed by DNA sequencing.

### Western Blot Analysis

For Western blot analysis, cells were lysed in lysis buffer. Cell lysates were separated by 7.5%–10% SDS-polyacrylamide gel electrophoresis (SDS-PAGE) and then transferred onto a nitrocellulose (NC) membrane. The membranes were sealed in phosphate-buffered saline with 0.1% Tween 20 (TBST) containing 5% non-fat dried milk for 45 min at room temperature (RT) and then were incubated with first antibodies at 4°C overnight. Next, the membranes were incubated with second antibodies for 45 min at RT. Finally, the membranes were detected with the Clarity™ Western ECL Substrate (Bio-Rad).

### Co-Immunoprecipitation

The cells were washed with pre-cold PBS for three times and lysed in RIPA lysis buffer (50 mM Tris–HCl (pH 7.4), 150 mM NaCl, 1% (vol/vol) NP-40, 1 mM EDTA, and 5% (vol/vol) glycerol) containing protease inhibitor cocktails. After 20 min, the lysed samples were centrifuged for 10 min at 4°C. A part of the lysates was saved as control. For immunoprecipitation, the rest of the lysates were incubated with the indicated antibodies at 4°C overnight and then incubated with protein G agarose for 2 h. The beads were washed for 5–7 times by RIPA washing buffer (50 mM Tris–HCl (pH 7.4), 300 mM NaCl, 1% (vol/vol) NP-40, 1 mM EDTA, and 5% (vol/vol) glycerol) and then reconstituted in 50 µl 2× SDS loading buffer. All targeted protein bands were immunoblotted with the indicated antibodies.

### Quantitative PCR

Total RNA was extracted with TRIzol reagent, following the manufacturer’s instructions. The mRNA was then used to create cDNA by using the M-MLV Reverse Transcriptase (Promega). Real-time quantitative RT-PCR was performed by using SYBR Green PCR Master Mix in the Roche LC480 following the manufacturer’s instructions. All real-time PCR primers were designed in Nucleotide of National Center for Biotechnology Information (NCBI). The following primers were used:

human GAPDH-F: 5′-AAGGCTGTGGGCAAGG-3′;

human GAPDH-R: 5′-TGGAGGAGTGGGTGTCG-3′;

human ASB17-F: 5′-CTGGGTTTTTGCCAGAAAAGGT-3′;

human ASB17-R: 5′-TGCCACTTAATGGGCTTGGA-3′;

human CCL2-F: 5′-GCTCAGCCAGATGCAATCAA-3′;

human CCL2-R: 5′-GACACTTGCTGCTGGTGATTC-3′;

human IL-6-F: 5′-ACCCCTGACCCAACCACAAAT-3′;

human IL-6-R: 5′-AGCTGCGCAGAATGAGATGAGTT-3′;

human IL-1β-F: CACGATGCACCTGTACGATCA;

human IL-1β-R: GTTGCTCCATATCCTGTCCCT;

human IP-10-F: GCCATTCTGATTTGCTGCCT;

human IP-10-R: TTGATGGCCTTCGATTCTGGA;

mouse GAPDH-F: 5′-TTCACCACCATGGAGAAGGC-3′;

mouse GAPDH-R: 5′-GGCATCGACTGTGGTCATGA-3′;

mouse CCL2-F: 5′-GACCCCAAGAAGGAATGGGT-3′;

mouse CCL2-R: 5′-ACCTTAGGGCAGATGCAGTT-3′;

mouse IL-6-F: 5′-CAACGATGATGCACTTGCAGA-3′;

mouse IL-6-R: 5′-TGACTCCAGCTTATCTCTTGGT-3′;

mouse ASB17-F: 5′-TAGTTAAGCGGCCCTCTCTG-3′;

mouse ASB17-R: 5′-GTCAAAGCCGTCCAAGTCAAC-3′;

Mouse IL-1β-F: CTGGTGTGTGACGTTCCCAT;

Mouse IL-1β-R:GTGGGTGTGCCGTCTTTCAT;

Mouse IP-10-F: ATGACGGGCCAGTGAGAATG;

Mouse IP-10-R:CGGATTCAGACATCTCTGCTCAT.

### Lentiviral Production and Infection

The pLenti-CMV vector was derived from the pLenti-CMV-GFP-Puro vector (Addgene, 17448). 3Flag-ASB17 (human) and 3Flag-Asb17 (mouse) were cloned and constructed into pLenti-CMV to generate lentiviruses. pLenti-3Flag-ASB17 (or pLenti-3Flag-Asb17), pMD2.G, and psPAX2 plasmids were co-transfected into HEK293T cells to generate lentivirus. HEK293T cell culture supernatants were harvested at 36 and 48 h after transfection. The culture supernatants were filtered through a 0.45-µm filter. THP-1 cells were infected with the lentivirus plus 8 μg/ml polybrene for 24–36 h. Next, 1 μg/ml puromycin was added into the culture supernatants for selection of THP-1 stably expressing ASB17 cells. After 5–7 days, THP-1 stably expressing ASB17 cells were identified by qPCR and immunoblot analysis.

### Immunofluorescence Microscopy

For immunofluorescence staining, the cells were washed three times with pre-cold PBS and fixed with 4% paraformaldehyde for 15 min. Then, the cells were permeabilized with PBS containing 0.5% Triton X-100 for 5 min and then blocked with PBS containing 5% bovine serum albumin (BSA) for 45 min at room temperature. Then, the cells were incubated with the indicated antibody at 4°C overnight, followed by incubation with anti-Mouse IgG DyLight 649 and anti-Rabbit IgG FITC at room temperature for 2 h. After washing three times, the cells were incubated with DAPI for 5 min in 37°C. Finally, the cells were analyzed using a confocal laser scanning microscope (FluoView FV1000; Olympus, Tokyo, Japan).

### *In Vivo* Ubiquitination Assay

Cells were lysed with 100 µl lyses buffer. After heating at 95°C for 5 min, lysates were diluted 10-fold with dilution buffer containing protease inhibitors. A part of the lysates was saved as input, and the rest of the lysates were immunoprecipitated with indicated antibodies. The rest of the procedures followed the Co-IP assays.

### Statistical Analyses

All experiments were reproducible and each set was repeated at least three times. For data with a normal distribution and homogeneity of variance, differences between two groups were statistically analyzed by a two-tailed Student’s *t*-test. Statistical significance was valued based on the *p* value. * indicates *p* < 0.05; ** indicates *p* < 0.01; and *** indicates *p* < 0.001. *p* < 0.05 was considered statistically significant.

## Results

### ASB17 Deficiency Inhibits LPS-Mediated NF-κB Activation in BMDCs

**Figure 1:**
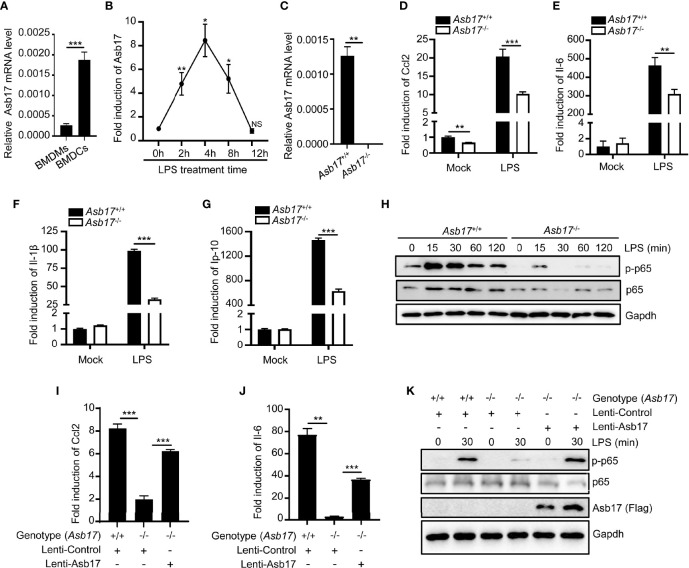
ASB17 deficiency inhibits LPS-mediated NF-κB activation in BMDCs. **(A)** BMDMs and BMDCs were isolated from wild-type mice; the mRNA expression of ASB17 in BMDMs and BMDCs were analyzed by qPCR. **(B)** BMDCs were isolated from wild-type mice and were stimulated by LPS (1 μg/ml) in different timepoints (0, 2, 4, 8, and 12 h), and the mRNA expression of ASB17 in BMDCs were analyzed by qPCR. **(C)** BMDCs were isolated from *ASB17* ^+/+^ and *ASB17* ^-/-^ mice, the mRNA expression of ASB17 in BMDCs were analyzed by qPCR. **(D–G)** BMDCs were isolated from *ASB17* ^+/+^ and *ASB17* ^-/-^ mice and were stimulated by LPS (1 μg/ml) for 0 and 2 h; the mRNA expression of CCL2 **(D)**, IL-6 **(E)**, IL-1β **(F)**, and IP-10 **(G)** in BMDCs were analyzed by qPCR. **(H)** BMDCs were isolated from *ASB17* ^+/+^ and *ASB17* ^-/-^ mice and were stimulated by LPS (1 μg/ml) in different timepoints (0, 15, 30, 60, and 120 min); the protein levels of p65 and phosph-p65 in BMDCs were analyzed by Western blotting. **(I, J)** BMDCs were isolated from *ASB17* ^+/+^ and *ASB17* ^-/-^ mice, and ASB17 was overexpressed in *ASB17* ^-/-^ BMDCs by infecting with the recombinant lentivirus. All cells were stimulated by LPS (1 μg/ml) for 0 or 2 h; the mRNA expressions of CCL2 and IL-6 in BMDCs were analyzed by qPCR. **(K)** BMDCs were isolated from *ASB17* ^+/+^ and *ASB17* ^-/-^ mice, and ASB17 was overexpressed in *ASB17* ^-/-^ BMDCs by infecting with the recombinant lentivirus. All cells were stimulated by LPS (1 μg/ml) for two timepoints (0 and 30 min); the protein levels of phosph-p65 in BMDCs were analyzed by Western blotting. Data are shown as means ± SD. *p < 0.05; **p < 0.01; ***p < 0.001.

The detection of Asb17 in mouse organs displayed that it mainly expressed in testis (Kim et al., 2004). However, the expression of this gene was detectable in bone marrow-derived macrophages (BMDMs) and dendritic cells (BMDCs) (**Figure 1A**). Especially, its expression level in BMDCs could be induced by LPS which was an important pyrogen derived from gram-negative bacteria (**Figure 1B**). These results indicated that ASB17 might have participated in pathological processes of infectious inflammation. We isolated BMDCs from *Asb17* ^-/-^ and wild-type mice (**Figure 1C**). We found that *Asb17* knocking-out significantly impaired the expression of the LPS-induced pro-inflammatory cytokines Ccl2, Il-6, Il-1β, and Ip-10 in BMDCs (**Figures 1D–G**). The NF-κB signal pathway plays a critical role in the production of the pro-inflammatory cytokines when TLR4 was activated by LPS (Guha and Mackman, 2001). By detecting this pathway, we found that Asb17 deficiency obviously decreased the phosphorylation of NF-κB p65 when BMDCs were stimulated with LPS (**Figure 1H**), indicating an impairment of NF-κB activation. In addition, we checked the induction of pro-inflammatory cytokines and the phosphorylation of NF-κB p65 when Asb17 was overexpressed in *Asb17* ^-/-^ BMDCs to verify its specificity. Asb17 overexpression in *Asb17* ^-/-^ BMDCs recovered the LPS-induced Ccl2 and Il-6 expression and NF-κB p65 activation (**Figures 1I–K**). Our results suggested that ASB17 was important for LPS-mediated NF-κB activation.

### Overexpression ASB17 Promotes LPS-Mediated NF-κB Activation in THP-1 Cells

**Figure 2:**
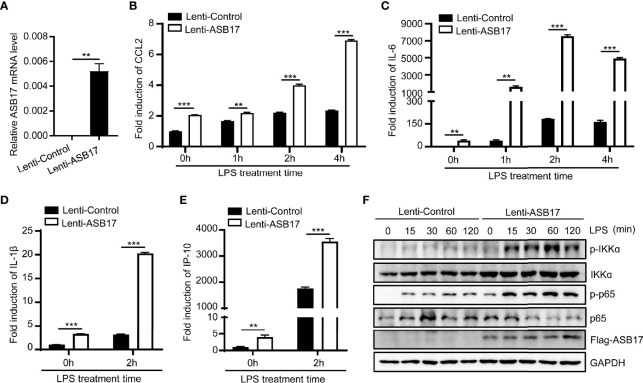
Overexpression ASB17 promotes LPS-mediated NF-κB activation in THP-1 cells. **(A)** Total RNAs were isolated from THP-1 stably expressing Flag-ASB17 and its control; mRNA levels of ASB17 in these cells were quantified by RT-PCR. **(B, C)** THP-1 stably expressing Flag-ASB17 and its control were stimulated by LPS (1 μg/ml) in different timepoints (0, 1, 2, and 4 h). The mRNA levels of IL-6 and CCL2 in these cells were quantified by RT-PCR. **(D, E)** THP-1 stably expressing Flag-ASB17 and its control were stimulated by LPS (1 μg/ml) in different timepoints (0 and 2 h). The mRNA levels of IL-1β and IP-10 in these cells were quantified by RT-PCR. **(F)** THP-1 stably expressing Flag-ASB17 and its control were stimulated by LPS (1 μg/ml) in different timepoints (0, 15, 30, 60, and 120 min); the protein levels of IKKα, phosph-IKKα, p65, and phosph-p65 in these cells were quantified by Western blotting. Data are shown as means ± SD. **p < 0.01; ***p < 0.001.

To further examine the role of ASB17 in NF-κB signaling, we constructed THP-1 cells stably expressing ASB17 protein (**Figure 2A**). Overexpression of ASB17 significantly promoted the expression of *IL-6*, *CCL2*, *IL-1β*, and *IP-10* whether with LPS stimulation or not (**Figures 2B–E**). Besides, ASB17 overexpression obviously enhanced the p65 and IKKα phosphorylation induced by LPS (**Figure 2F**). Overall, ASB17 could enhance LPS-mediated NF-κB activation.

### ASB17 Interacts With TRAF6

**Figure 3:**
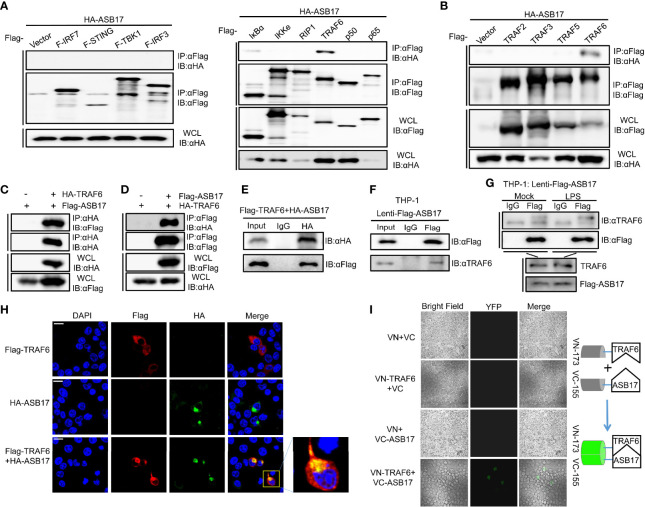
ASB17 interacts with TRAF6. ***(*A*)*** HEK293T cells were co-transfected with HA-ASB17 and Flag-IRF7, Flag-STING, Flag-TBK1, Flag-IRF3, Flag-IkBα, Flag-IKKe, Flag-RIP1, Flag-TRAF6, Flag-p50, or Flag-p65 in 6-cm cell dishes for 24–36 h, respectively. A part of the cell lysates as input and remaining cell lysates were immunoprecipitated with anti-Flag antibodies. **(B)** HEK293T cells were co-transfected with HA-ASB17 and Flag-TRAF2, Flag-TRAF3, Flag-TRAF5, or Flag-TRAF6 in 6-cm cell dishes for 24–36 h A part of the cell lysates as input and remaining cell lysates were immunoprecipitated with anti-Flag antibodies. **(C, D)** HEK293T cells were co-transfected with HA-TRAF6 and Flag-ASB17 in 10-cm cell dishes for 24–36 h A part of the cell lysates as input and remaining cell lysates were immunoprecipitated with anti-HA antibody **(C)** or anti-Flag antibody **(D)**. **(E)** HEK293T cells were co-transfected with Flag-TRAF6 and HA-ASB17 in 6-cm cell dishes for 24–36 h. A part of the cell lysates as input and remaining cell lysates were immunoprecipitated with IgG or anti-HA antibodies. **(F)** A part of THP-1 stably expressing ASB17 cell lysates as input and remaining cell lysates were immunoprecipitated with IgG or anti-Flag antibodies. **(G)** THP-1 stably expressing ASB17 cells were stimulated with LPS (1 μg/ml) in different timepoints (0 and 2 h) in 10-cm cell dishes. A part of the cell lysates as input and remaining cell lysates were immunoprecipitated with IgG or anti-Flag antibodies. **(H)** HEK293T cells were co-transfected with plasmids as indicated. Subcellular localizations of HA-ASB17 (green), Flag-TRAF6 (red), and nucleus marker DAPI (blue) were analyzed under confocal microscopy. Scale bar, 20 μm. **(I)** HEK293T cells were co-transfected with plasmids as indicated, biomolecular fluorescence complementation (BiFC) assays for detection of interactions between ASB17 and TRAF6. All bands were immunoblotted with the indicated antibodies.

To study how ASB17 regulated the NF-κB signal pathway, we screened key molecules involved in or related to this pathway including IRF7, STING, TBK1, IRF3, IκBα, IKKϵ, RIP1, TRAF6, P50, and P65 by immunoprecipitation to identify which of them might interact with ASB17. Among these molecules, we found that only TRAF6 was associated with ASB17 (**Figure 3A**). To assure that TRAF6 was associated with ASB17 truly and specifically, we also constructed TRAF family-related genes, TRAF2, TRAF3, and TRAF5, to perform the immunoprecipitation. The results indicated that TRAF6 could be precipitated by ASB17 specifically (**Figure 3B**). The further co-immunoprecipitation (Co-IP) and reciprocal Co-IP assays confirmed the interaction of ASB17 and TRAF6 (**Figures 3C–E**). To further confirm the interaction between ASB17 and TRAF6, we identified the interaction in THP-1 cells stably expressing ASB17 protein. The results indicated that ASB17 could interact with endogenous TRAF6 (**Figure 3F**). This interaction was enhanced by LPS treatment (**Figure 3G**). Confocal microscopy showed that ASB17 co-localized with TRAF6 in cells (**Figure 3H**). We also used the bimolecular fluorescence complementation (BiFC) analysis system to detect this protein–protein interaction. We constructed the VN-173-TRAF6 and VC-155-ASB17 plasmids and transfected them into cells. Fluorescence was not observed in control groups but was obviously observed in the experimental group in which VN-173-TRAF6 and VC-155-ASB17 were both transfected. The result suggests that ASB17 directly interacted with TRAF6 (**Figure 3I**). Overall, these results demonstrated that ASB17 was physically associated with TRAF6. Given that TRAF6 plays a key role in NF-κB signal transduction, we hypothesize that ASB17 regulated the NF-κB signal pathway *via* the interaction with TRAF6.

### ASB17 Suppresses TRAF6 Polyubiquitination and Stabilizes TRAF6 Protein

**Figure 4:**
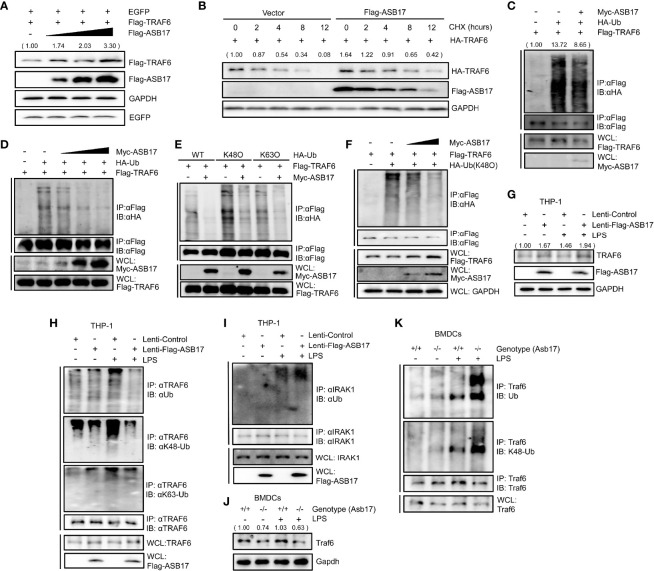
ASB17 suppresses TRAF6 polyubiquitination and stabilizes TRAF6 protein. **(A)** HEK293T cells were co-transfected with TRAF6, EGFP, and ASB17; the cell lysates were immunoblotted with indicated antibodies. **(B)** HEK293T cells were co-transfected with TRAF6 or ASB17, then were treated with CHX (50 μg/ml) in different timepoints; the cell lysates were immunoblotted with indicated antibodies. **(C)** HEK293T cells were transfected with TRAF6, Ub, or Flag-ASB17. A part of the cell lysates as input and remaining cell lysates were immunoprecipitated with anti-Flag antibody. **(D)** HEK293T cells were transfected with TRAF6, Ub, or a series of increasing amounts of Myc-ASB17 plasmids (0.5, 1, and 2 μg). A part of the cell lysates as input and remaining cell lysates were immunoblotted with anti-Flag antibodies. **(E)** HEK293T cells were transfected with TRAF6, ASB17, or Ub (WT, K48O, and K63O). A part of the cell lysates as input and remaining cell lysates were immunoprecipitated with anti-Flag antibody. **(F)** HEK293T cells were transfected with TRAF6, Ub (K48O), or a series of increasing amounts of Myc-ASB17 plasmids (1 and 2 μg). A part of the cell lysates as input and remaining cell lysates were immunoprecipitated with anti-Flag antibody and then immunoblotted with indicated antibodies. **(G–I)** THP-1 cells stably expressing ASB17 and control; the indicated THP-1 cells were stimulated by LPS for 0 and 2 h, and the cell lysates were immunoblotted with indicated antibodies **(G)**. A part of the cell lysates as input and the remaining cell lysates were immunoprecipitated with anti-TRAF6 antibody **(H)**. A part of the cell lysates as input and the remaining cell lysates were immunoprecipitated with anti-IRAK1 antibody **(I)**. **(J, K)** BMDCs were isolated from *ASB17* ^+/+^ and *ASB17* ^-/-^ mice, and cells were stimulated by LPS for 0 and 2 h. The cell lysates were immunoprecipitated with indicated antibody **(J)**. A part of the cell lysates as input and and remaining cell lysates were immunoprecipitated with anti-TRAF6 antibody **(K)**.

To explore how ASB17 affected the function of TRAF6, we investigated the TRAF6 protein levels. HEK293T were quantitatively transfected with TRAF6, EGFP (set as control), and different doses of ASB17. The Western-blotting analysis displayed that ASB17 increased the TRAF6 protein level but did not affect the EGFP (**Figure 4A**). To study the stability of TRAF6 protein, we performed a protein decay assay with cycloheximide (CHX) which blocked cellular protein synthesis. The results showed that ASB17 overexpression markedly reduced the decay rate of TRAF6 protein, indicating that ASB17 stabilized the TRAF6 protein (**Figure 4B**).

Then, we examined whether polyubiquitination of TRAF6 was regulated by ASB17. In HEK293T cells, ASB17 could markedly suppress the polyubiquitination of TRAF6 (**Figure 4C**). Moreover, ASB17 suppressed the polyubiquitination of TRAF6 in an ASB17 dose-dependent manner (**Figure 4D**). It had been reported that many E3 ubiquitin ligases targeted ubiquitin chains of linkages (K48-linked or K63-linked) to TRAF6 (Zhang et al., 2013; Wu et al., 2017). We constructed ubiquitin mutant vectors K48O and K63O (all lysine residues become arginine residues except its lysine residues at positions 48 and 63, respectively). The ubiquitin detection displayed that ASB17 could markedly suppress K48-linked polyubiquitination (**Figure 4E**). To further assure that ASB17 inhibited the K48-linked polyubiquitination of TRAF6, we showed the result that ASB17 could suppress K48-linked polyubiquitination of TRAF6 in an ASB17 dose-dependent manner (**Figure 4F**). Additionally, overexpression of ASB17 also elevated the endogenous TRAF6 protein level in THP-1 (**Figure 4G**). Overexpression of ASB17 significantly inhibited the TRAF6 polyubiquitination and K48-linked polyubiquitination in LPS-stimulated THP-1, but no obvious inhibition on the K63-linked polyubiquitination was observed when THP-1 was stimulated with LPS (**Figure 4H**). In order to confirm the specificity of ASB17-mediated TRAF6 deubiquitination by LPS stimulation, we verified whether the ubiquitination of IRAK1 could be altered by ASB17 after LPS stimulation. The results indicated that ASB17 did not reduce the ubiquitination of IRAK1 (**Figure 4I**). Consistent with this, Asb17 knocking-out reduced the endogenous Traf6 protein level and increased the Traf6 polyubiquitination in BMDCs (**Figures 4J, K**). Together, ASB17 could suppress the polyubiquitination of TRAF6, indicating that ASB17 protected TRAF6 from degradation to promote NF-kB activation.

### The aa177-250 Segment of ASB17 Is Required for the Interaction With the Zn Finger Domain of TRAF6 and Polyubiquitination Suppression of TRAF6

**Figure 5:**
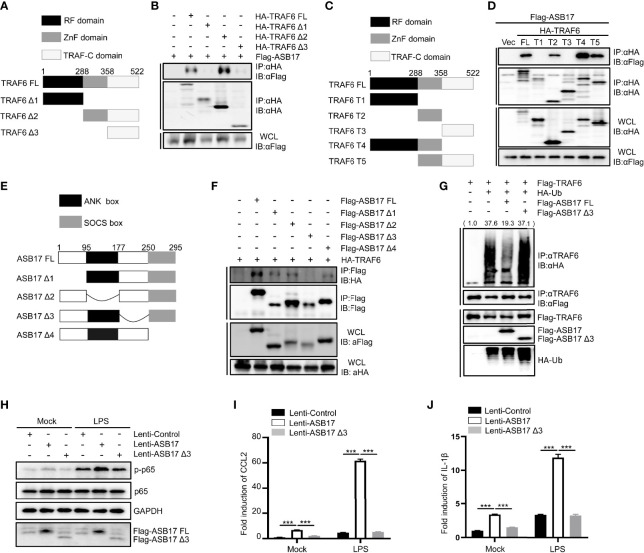
The aa177-250 segment of ASB17 is required for the interaction with the Zn finger domain of TRAF6 and polyubiquitination suppression of TRAF6. **(A)** Schematic diagram of TRAF6 and its truncated mutants (TRAF6 FL, TRAF6 Δ1, TRAF6 Δ2, and TRAF6 Δ3). **(B)** HEK293T cells were transfected with ASB17 and TRAF6 or its truncated mutants. A part of the cell lysates as input and remaining cell lysates were immunoprecipitated with anti-HA antibody. **(C)** Schematic diagram of TRAF6 and its truncated mutants (TRAF6 FL, TRAF6 T1, TRAF6 T2, TRAF6 T3, TRAF6 T4, and TRAF6 T5). **(D)** HEK293T cells were transfected with ASB17 and TRAF6 or its truncated mutants. A part of the cell lysates as input and remaining cell lysates were immunoprecipitated with anti-HA antibody. **(E)** Schematic diagram of TRAF6 and its truncated mutants (ASB17 FL, ASB17 Δ1, ASB17 Δ2, ASB17 Δ3, and ASB17 Δ4). **(F)** HEK293T cells were transfected with TRAF6 and ASB17 or its truncated mutants. A part of the cell lysates as input and remaining cell lysates were immunoblotted with anti-Flag antibodies. **(G)** HEK293T cells were transfected with TRAF6, Ub, ASB17, or ASB17 Δ3. A part of the cell lysates as input and remaining cell lysates were immunoprecipitated with anti-TRAF6 antibody. **(H)** THP-1 stably expressing ASB17, ASB17Δ3, and its control were stimulated by LPS (1 μg/ml) in different timepoints (0 and 2 h), and p65 and phosph-p65 in these cells were quantified by western blotting. **(I, J)** THP-1 stably expressing ASB17, ASB17Δ3, and its control were stimulated by LPS (1 μg/ml) in different timepoints (0 and 2 h). The mRNA levels of CCL2 and IL-1β in these cells were quantified by RT-PCR. Data are shown as means ± SD. ***p < 0.0001.

TRAF6 contains an RF domain, a ZnF domain, and a TRAF-C domain (**Figure 5A**). The Co-IP assay indicated that ASB17 could interact with the ZnF domain of TRAF6 (**Figure 5B**). In order to identify the interaction between ASB17 and the specific domain of TRAF6, we constructed more detailed domains (**Figure 5C**). The Co-IP assay also indicated that ASB17 could interact with the ZnF domain of TRAF6 (**Figure 5D**). It had been reported that ASB17 mainly contains the ANK box domain and SOCS box domain (**Figure 5E**). Co-IP assay indicated that the aa177–250 segment of ASB17 between the ANK box domain and the SOCS box domain was required for the interaction with TRAF6 (**Figure 5F**). To further study the effect of the ASB17 interaction on TRAF6 polyubiquitination, we performed the ubiquitin detecting assay with the ASB17 truncation. The results indicated that the truncation of the aa177–250 segment removed the polyubiquitination-suppressing activity of ASB17 on TRAF6 (**Figure 5G**). We also found that ASB17 could significantly enhance LPS-mediated NF-κB activation and LPS-induced pro-inflammatory cytokines CCL2 and IL-1β, but ASB17 Δ3 could not (**Figures 5H–J**). These data revealed that ASB17 *via* its aa177–250 segment interacted with TRAF6 to inhibit TRAF6 polyubiquitination.

### Hypothetical Model for the Role of ASB17 in the TRAF6-Mediated NF-κB Signal Pathway

**Figure 6:**
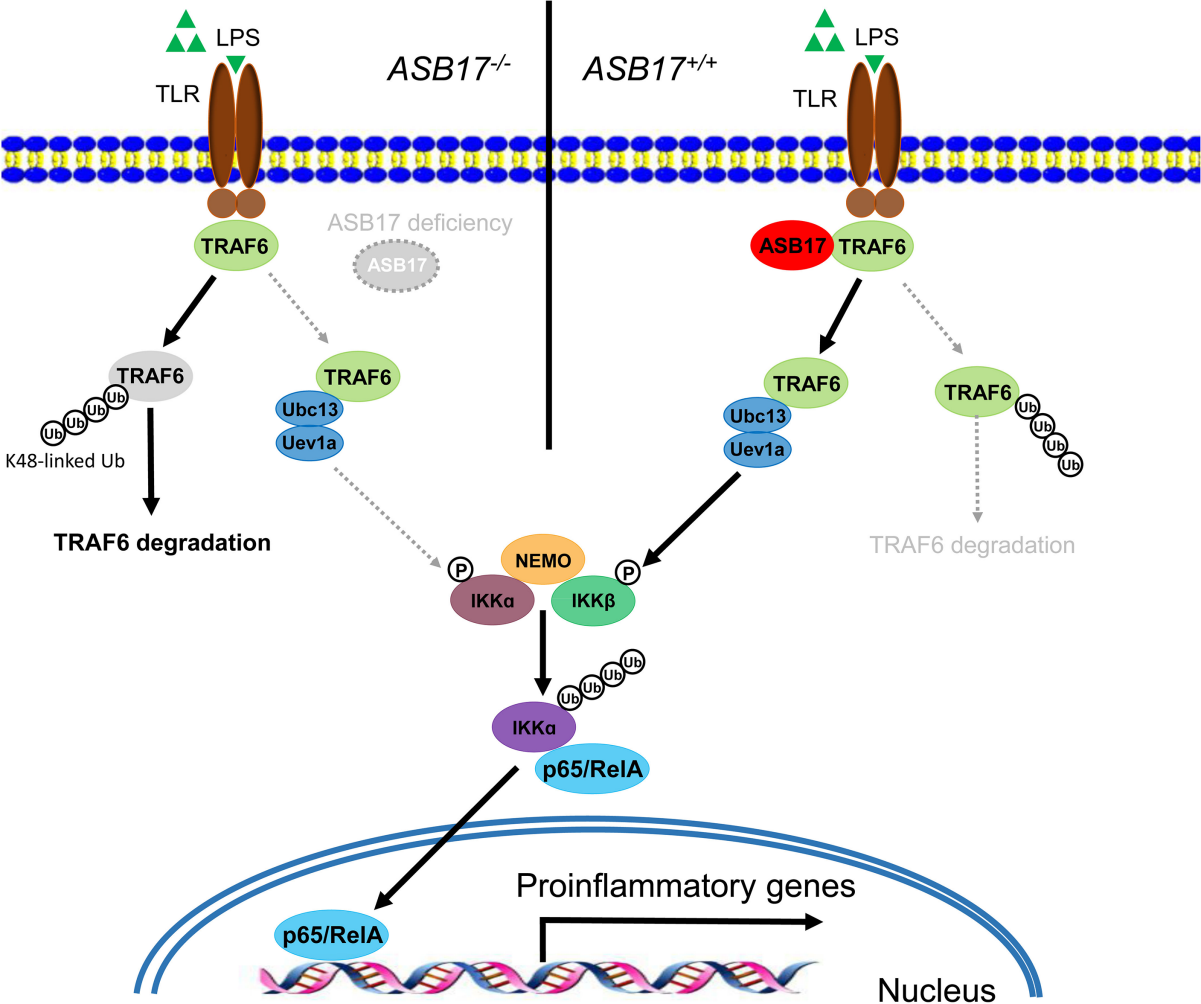
Hypothetical model for the role of ASB17 in the TRAF6-mediated NF-κB signal pathway. ASB17 interacts with TRAF6 and suppresses its K48-linked linked polyubiquitin, which protects TRAF6 from degradation. LPS activates NF-κB signaling through TRAF6 more effectively in wild-type DCs than in ASB17-knocking-out DCs.

ASB17 knocking-out in BMDCs impaired LPS-induced inflammatory cytokine expressions and markedly inhibited the activation of the NF-κB signal pathway. Overexpression of ASB17 elevated the levels of LPS-mediated NF-κB activation and the cytokine expressions in THP-1 cells. We found that ASB17 interacted with TRAF6 and suppressed K48-linked polyubiquitination of TRAF6, by which it stabilizes the TRAF6 protein. The TRAF6 partner role of ASB17 is crucial for LPS-mediated NF-κB activation and the burst of inflammation (**Figure 6**).

## Discussion

It is reported that ASB17 is mainly expressed in the testis, and we have found that ASB17 promotes testis cell apoptosis through specifically degrading BCLW and MCL1 (Yang et al., 2021). ASB17 may be a necessary gene for testis development and physiology. However, we found that ASB17 could be induced by LPS in dendritic cells. These indicated that ASB17 might be involved in immune and inflammatory responses. ASB17 promotes NF-κB activation and facilitated the expression of CCL2 and IL-6 when dendritic cells were stimulated with LPS. Thus, we screened the related factors involved in NF-κB signaling to search the target that ASB17 interacts with. We found that ASB17 was associated with TRAF6 and it significantly suppressed the K48-linked linked polyubiquitin of TRAF6. We suggested that ASB17 facilitates NF-κB activation through maintaining the TRAF6 protein stability.

Although ASB17 has E3 ubiquitin ligase activity like other members of the ASB family (Kohroki et al., 2005; Liu et al., 2019; Yang et al., 2021), our data reveal that it also functions as a ubiquitin-inhibiting factor. Therefore, its function is much more than ubiquitin ligase. It is suggested that ASB17 exerts bilateral functions in protein ubiquitination. We speculate that the function of ASB17 may be determined by the interaction site with the target protein. We previously reported that ASB17 induced the ubiquitination of BCLW and MCL1 for proteasomal degradation through its SOCS domain interaction with the two targets (Yang et al., 2021). The segment aa177–aa250 of ASB17 is required for the association with TRAF6 in the immunoprecipitation assay. The biological function of this segment is unclear before. It is between ankyrin repeat and SOCS domains. We define it as a TRAF6-associated domain and speculate that it mediates the ubiquitination-inhibiting activity of ASB17. In fact, it is not surprising that a protein has both deubiquitination and ubiquitination activities. The amino-terminal domain of A20, as a de-ubiquitinating enzyme, removes lysine-63 (K63)-linked ubiquitin chains from the receptor-interacting protein (RIP), while the carboxy-terminal domain of A20 functions as a ubiquitin ligase by promoting RIP K48-linked polyubiquitination for proteasomal degradation (Wertz et al., 2004). Although ASB17 does not contain a DUB domain, it may regulate ubiquitination of other protin by recruiting an E3 ligase to block ubiquitination of TRAF6, or recruit a de-ubiquitinase to de-ubiquitinate TRAF6.

Actually, there is faultiness in this study. Because of TRAF6 as the target of ASB17 we screened out from a few candidates which was not a global screening, ASB17 might regulate NF-κB signaling through other targets. Thus, it is necessary to knock out TRAF6 in mice or immune cell lines in the future to look into this possibility. Besides, we did not study the role of ASB17 in certain infectious inflammation models, as we focused on the cellular mechanism of NF-κB activation involved in pro-inflammatory cytokine induction. We revealed the biological role of ASB17 in TRAF6/NF-κB signaling. Given the importance of its target TRAF6 in cells, it is worth to study the functions of ASB17 in physiology and pathology by using clinical or animal models.

Therefore, we report a novel function of ASB17 that it targets TRAF6 to suppress its K48-linked polyubiquitination and proteasomal degradation. Besides, we recognize the important role of ASB17 in inflammation that it enhances NF-κB activation by maintaining TRAF6 stability.

## Data Availability Statement

The original contributions presented in the study are included in the article/supplementary material. Further inquiries can be directed to the corresponding authors.

## Ethics Statement

The animal study was reviewed and approved by the Institutional Animal Care and Use Committee (IACUC) of the College of Life Sciences, Wuhan University.

## Author Contributions

Conceptualization, PW, GY, YL, and JW. Data curation, GY and SZ. Formal analysis, PW, GY, SZ, YZ, YJ, XC, ZL, and PP. Funding acquisition, YL and JW. Investigation, GL, XlC, QZ, WZ, and QT. Methodology, PW, GY, and SZ. Project administration, YL and JW. Software, ZL, PP, GL, XlC, and QZ. Supervision, JW. Validation, ZL, PP, GL, XlC, and QZ. Visualization, PW, GY, and SZ. Writing—original draft, PW, GY, and SZ. Writing—review and editing, YL and JW. All authors contributed to the article and approved the submitted version.

## Funding

This work was supported by the National Natural Science Foundation of China (81730061, 32070148, and 81471942), the Guangdong Province “Pearl River Talent Plan” Innovation and Entrepreneurship Team Project (2017ZT07Y580), the Guangdong Basic and Applied Basic Research Foundation (2020A1515010369, 2019A1515011073), and the Fundamental Research Funds for the Central Universities (21620401).

## Conflict of Interest

The authors declare that the research was conducted in the absence of any commercial or financial relationships that could be construed as a potential conflict of interest.

## Publisher’s Note

All claims expressed in this article are solely those of the authors and do not necessarily represent those of their affiliated organizations, or those of the publisher, the editors and the reviewers. Any product that may be evaluated in this article, or claim that may be made by its manufacturer, is not guaranteed or endorsed by the publisher.

## References

[B1] Cao C. An R. Yu Y. Dai H. Qu Z. Gao M.. (2019). BICP0 Negatively Regulates TRAF6-Mediated NF-kappaB and Interferon Activation by Promoting K48-Linked Polyubiquitination of TRAF6. Front. Microbiol. 10, 3040. doi: 10.3389/fmicb.2019.03040 31969874 PMC6960150

[B2] Cao Z. Xiong J. Takeuchi M. Kurama T. Goeddel D. V. (1996). TRAF6 Is a Signal Transducer for Interleukin-1. Nature 383, 443–446. doi: 10.1038/383443a0 8837778

[B3] Chen F. Y. Huang M. Y. Lin Y. M. Ho C. H. Lin S. Y. Chen H. Y.. (2019). BIK Ubiquitination by the E3 Ligase Cul5-ASB11 Determines Cell Fate During Cellular Stress. J. Cell Biol. 218, 3002–3018. doi: 10.1083/jcb.201901156 31387940 PMC6719446

[B4] Chung J. Y. Lu M. Yin Q. Lin S. C. Wu H. (2007). Molecular Basis for the Unique Specificity of TRAF6. Adv. Exp. Med. Biol. 597, 122–130. doi: 10.1007/978-0-387-70630-6_10 17633022

[B5] Dainichi T. Matsumoto R. Mostafa A. Kabashima K. (2019). Immune Control by TRAF6-Mediated Pathways of Epithelial Cells in the EIME (Epithelial Immune Microenvironment). Front. Immunol. 10, 1107. doi: 10.3389/fimmu.2019.01107 31156649 PMC6532024

[B6] Deng L. Wang C. Spencer E. Yang L. Braun A. You J.. (2000). Activation of the IkappaB Kinase Complex by TRAF6 Requires a Dimeric Ubiquitin-Conjugating Enzyme Complex and a Unique Polyubiquitin Chain. Cell 103, 351–361. doi: 10.1016/S0092-8674(00)00126-4 11057907

[B7] Du M. Yuan L. Tan X. Huang D. Wang X. Zheng Z.. (2017). The LPS-Inducible lncRNA Mirt2 Is a Negative Regulator of Inflammation. Nat. Commun. 8, 2049. doi: 10.1038/s41467-017-02229-1 29230038 PMC5725456

[B8] Gabay C. Lamacchia C. Palmer G. (2010). IL-1 Pathways in Inflammation and Human Diseases. Nat. Rev. Rheumatol. 6, 232–241. doi: 10.1038/nrrheum.2010.4 20177398

[B9] Guha M. Mackman N. (2001). LPS Induction of Gene Expression in Human Monocytes. Cells Signal. 13, 85–94. doi: 10.1016/S0898-6568(00)00149-2 11257452

[B10] Guo J. H. Saiyin H. Wei Y. H. Chen S. Chen L. Bi G.. (2004). Expression of Testis Specific Ankyrin Repeat and SOCS Box-Containing 17 Gene. Arch. Androl. 50, 155–161. doi: 10.1080/01485010490425485 15204681

[B11] Hayden M. S. Ghosh S. (2014). Regulation of NF-kappaB by TNF Family Cytokines. Semin. Immunol. 26, 253–266. doi: 10.1016/j.smim.2014.05.004 24958609 PMC4156877

[B12] Kawai T. Akira S. (2010). The Role of Pattern-Recognition Receptors in Innate Immunity: Update on Toll-Like Receptors. Nat. Immunol. 11, 373–384. doi: 10.1038/ni.1863 20404851

[B13] Kim K. S. Kim M. S. Kim S. K. Baek K. H. (2004). Murine Asb-17 Expression During Mouse Testis Development and Spermatogenesis. Zygote 12, 151–156. doi: 10.1017/S0967199404002722 15460110

[B14] Kohroki J. Nishiyama T. Nakamura T. Masuho Y. (2005). ASB Proteins Interact With Cullin5 and Rbx2 to Form E3 Ubiquitin Ligase Complexes. FEBS Lett. 579, 6796–6802. doi: 10.1016/j.febslet.2005.11.016 16325183

[B15] Kwon S. Kim D. Rhee J. W. Park J. A. Kim D. W. Kim D. S.. (2010). ASB9 Interacts With Ubiquitous Mitochondrial Creatine Kinase and Inhibits Mitochondrial Function. BMC Biol. 8, 23. doi: 10.1186/1741-7007-8-23 20302626 PMC2852384

[B16] Lamothe B. Campos A. D. Webster W. K. Gopinathan A. Hur L. Darnay B. G. (2008). The RING Domain and First Zinc Finger of TRAF6 Coordinate Signaling by Interleukin-1, Lipopolysaccharide, and RANKL. J. Biol. Chem. 283, 24871–24880. doi: 10.1074/jbc.M802749200 18617513 PMC2529010

[B17] Liu P. Verhaar A. P. Peppelenbosch M. P. (2019). Signaling Size: Ankyrin and SOCS Box-Containing ASB E3 Ligases in Action. Trends Biochem. Sci. 44, 64–74. doi: 10.1016/j.tibs.2018.10.003 30446376

[B18] Lv Y. Kim K. Sheng Y. Cho J. Qian Z. Zhao Y. Y.. (2018). YAP Controls Endothelial Activation and Vascular Inflammation Through Traf6. Circ. Res. 123, 43–56. doi: 10.1161/CIRCRESAHA.118.313143 29794022 PMC6014930

[B19] Matsumoto R. Dainichi T. Tsuchiya S. Nomura T. Kitoh A. Hayden M. S.. (2018). Epithelial TRAF6 Drives IL-17-Mediated Psoriatic Inflammation. JCI Insight 3 (15), e121175. doi: 10.1172/jci.insight.121175 PMC6129131 30089718

[B20] Uematsu K. Okumura F. Tonogai S. Joo-Okumura A. Alemayehu D. H. Nishikimi A.. (2016). ASB7 Regulates Spindle Dynamics and Genome Integrity by Targeting DDA3 for Proteasomal Degradation. J. Cell Biol. 215, 95–106. doi: 10.1083/jcb.201603062 27697924 PMC5057283

[B21] Wan P. Zhang Q. Liu W. Jia Y. Ai S. Wang T.. (2019). Cullin1 Binds and Promotes NLRP3 Ubiquitination to Repress Systematic Inflammasome Activation. FASEB J. 33, 5793–5807. doi: 10.1096/fj.201801681R 30653357

[B22] Wertz I. E. O'rourke K. M. Zhou H. Eby M. Aravind L. Seshagiri S.. (2004). De-Ubiquitination and Ubiquitin Ligase Domains of A20 Downregulate NF-KappaB Signalling. Nature 430, 694–699. doi: 10.1038/nature02794 15258597

[B23] Wu Y. Hu Y. Wang B. Li S. Ma C. Liu X.. (2020). Dopamine Uses the DRD5-ARRB2-PP2A Signaling Axis to Block the TRAF6-Mediated NF-KappaB Pathway and Suppress Systemic Inflammation. Mol. Cell 78, 42–56.e46. doi: 10.1016/j.molcel.2020.01.022 32035036

[B24] Wu C. Su Z. Lin M. Ou J. Zhao W. Cui J.. (2017). NLRP11 Attenuates Toll-Like Receptor Signalling by Targeting TRAF6 for Degradation via the Ubiquitin Ligase RNF19A. Nat. Commun. 8, 1977. doi: 10.1038/s41467-017-02073-3 29215004 PMC5719394

[B25] Yang G. Wan P. Xiang Q. Huang S. Huang S. Wang J.. (2021). E3 Ubiquitin Ligase ASB17 Promotes Apoptosis by Ubiquitylating and Degrading BCLW and MCL1. Biol. (Basel) 10 (3), 234. doi: 10.3390/biology10030234 PMC8003104 33803505

[B26] Zhang X. Zhang J. Zhang L. Van Dam H. Ten Dijke P. (2013). UBE2O Negatively Regulates TRAF6-Mediated NF-KappaB Activation by Inhibiting TRAF6 Polyubiquitination. Cell Res. 23, 366–377. doi: 10.1038/cr.2013.21 23381138 PMC3587711

[B27] Zhao W. Wang L. Zhang M. Yuan C. Gao C. (2012). E3 Ubiquitin Ligase Tripartite Motif 38 Negatively Regulates TLR-Mediated Immune Responses by Proteasomal Degradation of TNF Receptor-Associated Factor 6 in Macrophages. J. Immunol. 188, 2567–2574. doi: 10.4049/jimmunol.1103255 22323536

